# Reliability Evaluation for Clustered WSNs under Malware Propagation

**DOI:** 10.3390/s16060855

**Published:** 2016-06-10

**Authors:** Shigen Shen, Longjun Huang, Jianhua Liu, Adam C. Champion, Shui Yu, Qiying Cao

**Affiliations:** 1 Department of Computer Science and Engineering, Shaoxing University, Shaoxing 312000, China; hlj_jlh@163.com; 2 College of Mathematics, Physics and Information Engineering, Jiaxing University, Jiaxing 314001, China; ljh_541@163.com; 3 Department of Computer Science and Engineering, The Ohio State University, Columbus, OH 43210, USA; champion@cse.ohio-state.edu; 4 School of Information Technology, Deakin University, Burwood 3125, Australia; syu@deakin.edu.au; 5 College of Computer Science and Technology, Donghua University, Shanghai 201620, China; caoqiying@dhu.edu.cn

**Keywords:** wireless sensor network, reliability evaluation, malware propagation, epidemic theory, continuous-time Markov chain, reliability theory

## Abstract

We consider a clustered wireless sensor network (WSN) under epidemic-malware propagation conditions and solve the problem of how to evaluate its reliability so as to ensure efficient, continuous, and dependable transmission of sensed data from sensor nodes to the sink. Facing the contradiction between malware intention and continuous-time Markov chain (CTMC) randomness, we introduce a strategic game that can predict malware infection in order to model a successful infection as a CTMC state transition. Next, we devise a novel measure to compute the Mean Time to Failure (MTTF) of a sensor node, which represents the reliability of a sensor node continuously performing tasks such as sensing, transmitting, and fusing data. Since clustered WSNs can be regarded as parallel-serial-parallel systems, the reliability of a clustered WSN can be evaluated via classical reliability theory. Numerical results show the influence of parameters such as the true positive rate and the false positive rate on a sensor node’s MTTF. Furthermore, we validate the method of reliability evaluation for a clustered WSN according to the number of sensor nodes in a cluster, the number of clusters in a route, and the number of routes in the WSN.

## 1. Introduction

Wireless Sensor Networks (WSNs) play an important role in daily life, as numerous modern information systems rely on WSNs, which consist of many sensor nodes with limited computation, storage, and communication resources. WSN applications include environmental, highway, and patient health monitoring as well as other commercial uses [1]. To realize these applications, the research community has focused on ensuring the reliability of WSNs. By definition, *reliability* reflects the ability of a system or component to perform its required functions under stated conditions for a specified period of time. Due to the nature of data collection in WSNs, this ability has been a major challenge in applying WSNs for successful monitoring. All sensor nodes need to send their sensed data towards the sink. Hence, packet loss due to transmission errors, packet collisions, interference, node failures, and malicious attacks is common [2]. Therefore, reliability evaluation for WSNs is vital in order to guarantee the delivery of sensed data from sensor nodes to the sink. In addition, reliability evaluation is crucial for maintaining sensor nodes’ functionality as nodes face attacks from self-replicating malicious code (*malware* for short).

In practice, WSNs are prone to malware propagation [3] for two reasons. First, sensor nodes have similar hardware and software, which results in large-scale malware propagation if even one sensor node is compromised. Second, there are several over-the-air reprogramming protocols (such as Trickle, Firecracker, Deluge, and MNP) that reconfigure sensor nodes without physical contact. Such protocols provide an opportunity for malware to spread across WSNs. Hence, WSNs’ reliability needs to be evaluated under epidemic-malware propagation conditions. This article focuses on such evaluation.

Epidemic models are borrowed from epidemiology to describe malware propagation in WSNs, since there are strong similarities between biological viruses’ self-replication and malware propagation. Classical epidemic models that have attracted much attention in the scientific community are classified among several families. The first family, the Susceptible-Infected (SI) model, is suitable for situations where nodes are either susceptible or infected. In this model, the state transition of any node is only from state *Susceptible* (*S*) to state *Infected* (*I*) and it is assumed that infected nodes remain in state *I* forever. The second family, the Susceptible-Infected-Susceptible (SIS) model, considers a susceptible node’s infection with a certain probability when it comes into contact with another infected node. Unlike the SI model, an infected node can be removed with a different probability, becoming susceptible again. The third family, the Susceptible-Infected-Removed (SIR) model, adds the state *Removed* (*R*) by extending the SI model. In this model, a susceptible node can be infected only once, since a node transforming from state *I* to state *R* becomes immune and is hence unable to propagate malware to other susceptible nodes.

A continuous-time Markov chain (CTMC) provides considerable flexibility for modeling state transitions of sensor nodes, which is suitable for illustrating malware propagation. In general, when the CTMC is used, it includes a set of discrete states and is formally described by a state-transition-rate diagram. The diagram indicates possible states of a sensor node along with directed arcs that characterize transition rates between states. Since malware actions lead to state transitions in a CTMC, it shows the process of malware propagation.

However, the CTMC, which is only one type of stochastic model, is insufficient for correctly treating malware infections that often result in security failures for sensor nodes. Malware residing in an infected sensor node always intentionally propagates itself to other susceptible sensor nodes; thus, such infections cannot be modeled as a stochastic process. Even if the time to propagate malware may be randomly distributed, the decision to propagate is not. To solve this problem, we are motivated to leverage a strategic game to obtain the malware’s expected propagation probability.

Game theory investigating strategic decision-making among players has been widely employed in the field of WSN security [4,5,6,7,8,9,10,11,12] such as optimizing intrusion detection strategies [5,6,10,11,12], securing data aggregation [7], localizing malicious nodes [8], and providing secure defenses for virtual sensor services [9]. This efficient mathematical tool is also suitable to explore different critical decisions during malware propagation. Usually, WSNs guard susceptible sensor nodes from malware attacks using Intrusion Detection Systems (IDSes). However, this method inevitably increases nodes’ costs upon launching IDS agents, since sensor nodes have limited computational resources. Malware achieves greater gains by infecting more sensor nodes and eavesdropping on sensed data from infectious nodes, but this infection behavior obviously increases the probability of detection by IDSes. Thus, malware selects an optimal infection strategy to determine its propagation.

In this paper, we study reliability evaluation for clustered WSNs under epidemic-malware propagation. We use a strategic game to predict malware’s infection behavior whose consequences we integrate into the transition probability upon infection of a sensor node. As a result, we determine how to relate the intent of malware infection to the CTMC’s randomness. Next, we propose a novel measure to reflect the reliability of a sensor node. After considering clustered WSNs as parallel-serial-parallel systems due to their communication modes, we obtain equations to compute the reliability of a cluster, a route, and a clustered WSN, respectively.

Our main contributions are as follows:

(1) We relate the intent of malware infection to the CTMC’s randomness by introducing a strategic game that can predict malware’s infection behavior. In this manner, state transitions of a sensor node that arise from malware actions can be modeled by the CTMC; and
(2) We propose a novel measure to compute the Mean Time to Failure (MTTF) of a sensor node, which represents the reliability of a sensor node continuously performing tasks such as sensing, transmitting, and fusing data. Thus, we can deduce the reliability of a cluster, a route, and a clustered WSN from the perspective of a parallel-serial-parallel system, respectively. This method of reliability evaluation for clustered WSNs under epidemic-malware propagation can help establish theoretical foundations that guide rules for applying reliability techniques. Consequently, WSNs that guarantee reliable delivery of sensor nodes’ sensed data may be realized.

The rest of this article is organized as follows: we first review related work and highlight the salient features of our approach in Section 2. We describe infections as state transitions from the view of a CTMC in Section 3. We obtain the infection probability by introducing a strategic malware-infection game in Section 4. We propose measures of reliability evaluation for clustered WSNs under the scenario of epidemic-malware propagation in Section 5. We validate the proposed measures’ efficacy in Section 6. Finally, we conclude the article in Section 7.

## 2. Related Work

Based on classical epidemic models, many extended studies have been performed to describe the characteristics of malware propagation in WSNs. In a good survey, Yu *et al.* [13] presented current works on modeling malware propagation. Generally, sensor nodes periodically enter sleep mode to save energy. Typical models reflecting nodes sleeping during malware propagation include EiSIRS [14], a modified SI model [15], a modified SIS model [16], and Shen’s model [17]. Moreover, sensor nodes “die” due to energy exhaustion or intentional destruction by malware. Thus, a dead state was introduced in iSIRS [18] based on the SIR model and Shen’s model [17]. Furthermore, a reaction-diffusion-theoretic model [19], a pulse-differential-equation-based SIR model [20], and a susceptible-exposed-infected-recovered-susceptible model [21] were proposed in order to foresee spatial distribution and the temporal dynamics characteristic of malware propagation. In addition, Yu *et al.* [22] proposed a two-layer malware propagation model that better represents malware propagation in large-scale networks compared with existing single-layer epidemic models. Keshri and Mishra [23] presented a susceptible-exposed-infectious-recovered model with two time delays for charactering the transmission dynamics of malware propagation. Zhu and Zhao [24] explored a SIR-based nonlinear malware propagation model in WSNs. Wang *et al.* [25] presented a survey on modeling malware propagation in networks including WSNs. Other typical models [26,27,28,29] address the problem of malware propagation in multi-hop networks, which can help illustrate malware propagation in WSNs.

Several authors have considered decision-making dilemmas arising during malware propagation. Khouzani *et al.* [30] established a zero-sum dynamic game between the network system and the malware, given that malware can dynamically alter infection parameters based on the network system’s dynamics. Jin *et al.* [31] used an evolutionary game to construct a malware propagation model under bounded rationality, where the game is to predict the trend of malware’s evolutionary infection. Spyridopoulos *et al.* [32] employed a complete information game to obtain the defender’s optimal strategy that minimizes the security cost as well as the malware effect. In addition, Trajanovski *et al.* [33] found decentralized optimal protection strategies for the network system by proposing a game-theoretic framework and seeking its Nash equilibria and the Price of Anarchy.

Several methods have been proposed using various techniques to cope with the challenge of evaluating WSNs’ reliability. In a pioneering work [34], the authors employed a probabilistic graph to represent WSNs given a failure probability estimation of sensor nodes. Kar *et al.* [35] modeled WSNs’ energy reliability assuming Markovian sensor discharge/recharge periods. Distefano [36] used dynamic reliability block diagrams to represent static structural interactions between sensor nodes, where sleep/wake-up standby policies and interference are considered dynamically. Based on [36], he further gave a reliability evaluation model integrating Petri nets [37]. Silva *et al.* [38] proposed an evaluation methodology supporting arbitrary failure conditions based on automatically generated fault trees. Niyato *et al.* [39] proposed reliability analysis of wireless communications systems in the smart grid, which is also suitable for WSNs. Kamal *et al.* [40] developed a novel framework called Packet-Level Attestation for sensor data reliability evaluation using the spatial relationship among data sensed by nearby sensor nodes. In [41] Dâmaso *et al.* proposed a reliability evaluation model based on routing algorithms in WSNs and sensor nodes’ various battery levels. According to MAC protocols adopted in WSNs, Wang *et al.* [42] evaluated a sensor node’s reliability under three typical working scenarios including sensor nodes always in active mode, alternating between sleep and active modes on average, and alternating between these modes based on a certain distribution. Zonouz *et al.* [43] evaluated the reliability of energy harvesting sensor nodes and battery-powered sensor nodes as well as develop the corresponding wireless-link-reliability models. Cai *et al.* [44] characterized event-driven WSNs according to limited node battery energy and shadowing under channel fading, obtaining reliable data flows in WSNs via wireless link reliability and node energy availability. Wang *et al.* [45] analyzed a body sensor node’s reliability subject to probabilistic competition between propagation effects and probabilistic failure isolation. Yan *et al.* [46] proposed a symbolic ordered-binary-decision-diagram-multicast method to evaluate the reliability of multicast WSNs. Zhu *et al.* [47] proposed mission-oriented and transmission-paths-based models for evaluating WSNs’ transmission reliability. Other measures closely related to reliability include dependability and survivability such as the stochastic-activity-network-based dependability measure [48], the epidemic-theory-based survivability measure [49,50], survivability analysis using probabilistic model checking [51], and natural-tenacity-based survivability evaluation for mobile WSNs [52].

Unlike this body of work, to the best of our knowledge this work is the first that concentrates on reliability evaluation for clustered WSNs under epidemic-malware propagation conditions. While our epidemic model is similar to [14,17], we further model all state transitions of a sensor node as a CTMC by integrating the malware’s infection probability predicted by our strategic malware-infection game into the transition probability. We apply the approach proposed in [53] to compute the MTTF from a Markov process; however, we further find the equation to compute the reliability of a sensor node under epidemic-malware propagation, which is a novel measure. Considering the topology of a typical clustered WSN, we can thus deduce the reliability of a cluster, a route, and a clustered WSN based on reliability theory. In summary, we propose a unified framework for malware-infection and reliability evaluation that integrates both security and reliability properties of a clustered WSN during the evaluation process.

## 3. Modeling State Transitions of a Sensor Node as a CTMC

**Figure 1:**
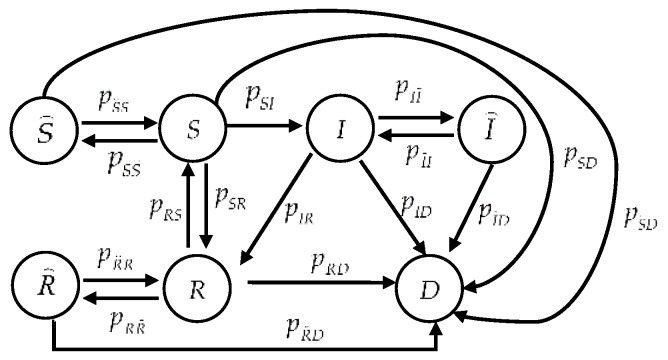
A CTMC that illustrates the state transition of a sensor node.

The various epidemic-malware propagation models mentioned above are actually state transition models. These states are mutually exclusive: a sensor node is in exactly one state at any time. During its lifecycle, a sensor node interchanges among different states. Figure 1 illustrates a CTMC indicating state transitions of a sensor node, where $p_{ij}$, $i,\;j\in \{S,\;\overset{⌢}{S},\;I,\;\overset{⌢}{I},\;R,\;\overset{⌢}{R},\;D\}$, denotes the transition rate from state i to state j, and S, $\overset{⌢}{S}$, I, $\overset{⌢}{I}$, R, $\overset{⌢}{R}$, and D denote states *Susceptible*, *Susceptible* while *sleeping*, *Infected*, *Infected* while *sleeping*, *Recovered*, *Recovered* while *sleeping*, and *Dead*, respectively. Each sensor node’s characteristics determine its state. State S denotes a sensor node that works normally and is not infected by malware, but it is susceptible to malware. $\overset{⌢}{S}$ denotes a sleeping sensor node that malware cannot infect although the node is susceptible. I denotes a sensor node that has been infected by malware and may propagate malware to neighboring nodes with which it communicates as it is under the malware’s control. $\overset{⌢}{I}$ denotes an infected sensor node that is sleeping; thus, it cannot propagate malware. R denotes a “recovered” node that can be “immunized” to current malware, but not to future malware. $\overset{⌢}{R}$ denotes a recovered node that is sleeping. D denotes a node that is unusable because malware exhausted its energy, deliberately destroyed it, or both.

In practice, behaviors of both sensor nodes and malware trigger each state transition. For each sensor node, installing security patches is a normal method to either prevent susceptible nodes from known malware by fixing bugs or to remedy an infected node and immunize it to known malware. Thus, this action results in the state transitions S→R and I→R. In general, each node is scheduled to sleep, which saves energy, or to awaken, which leads to the state transitions $S\to \overset{⌢}{S}$ or $\overset{⌢}{S}\to S$. The state transitions $I\to \overset{⌢}{I}$ or $\overset{⌢}{I}\to I$ as well as $R\to \overset{⌢}{R}$ or $\overset{⌢}{R}\to R$ follow likewise. When confronting unknown malware, a sensor node usually lacks immunity, and the state transition R→S follows. In addition, the state transitions S→D, $\overset{⌢}{S}\to D$, I→D, $\overset{⌢}{I}\to D$, R→D, and $\overset{⌢}{R}\to D$ take place when malware exhausts a sensor node’s energy. On the other hand, contamination by malware leads to the state transition S→I. Moreover, malware can deliberately destroy an infectious sensor node besides the case of death from energy exhaustion; hence, the state transition I→D occurs.

However, the state transition S→I cannot be formulated as a stochastic process because the infection behavior resulting in this transition is deliberate. Even though the time to execute action *Infect* is stochastically distributed, the decision to execute the actual infection is not. Since malware decides whether to infect a susceptible sensor node, there is an infection probability that malware determines to execute infection. We let $\rho_{I}$ be the probability that the malware will choose action *Infect* to propagate itself and λ be the probability of a successful infection in order to formalize the malware’s decision. Thus, the infection rate (*i.e.*, the state transition rate) from state S to state I becomes:

(1) $$p_{SI}=\rho_{I}\lambda$$

By introducing the infection probability $\rho_{I}$, we can model the consequence of a successful infection as one deliberate state transition of the CTMC, which describes the dynamics of a sensor node’s behavior. In this manner, our modeling approach concentrates on the higher-level effects of infection on a sensor node rather than the lower-level specific infection procedures.

## 4. A Strategic Malware-Infection Game to Obtain the Infection Probability

We employ a strategic game to predict the malware’s infection behavior, namely obtaining the probability $\rho_{I}$. We adopt game-theoretic analysis as the following dilemma arises: the malware attempts to infect as many sensor nodes as possible without detection, whereas the defender attempts to enhance the network robustness by detecting more malware. Facing this dilemma, we introduce players *malware* and *system* to play the strategic game. Even though there are various kinds of malware whose goals may be to eavesdrop on private sensed data or to disable communication among sensor nodes, it is sufficient to regard all malware as player *malware* due to malware’s similar motivations and skills. Player *system*, the opponent of *malware*, actually corresponds to IDSes residing in WSNs.

In practice, iterations of the strategic game can be depicted as follows. There are discrete periods of time in which player *malware* launches infection. Player *system* intends to prevent infection in any of these periods. In each period, each player has two actions: player *malware* can either infect or not infect while player *system* can either defend or not defend. But each player can choose only one action with either pure or mixed strategies. If player *malware* takes no action and player *system* does not defend, then the game enters the next stage. Next, we formally define our strategic game and we explore the game entirely from *malware*’s view, since the target of our strategic game is to predict the infection intention of player *malware* and not to obtain the optimal defense strategies for player *system*.

**Definition 1**.  *The Strategic Malware-Infection Game (SMIG) is formulated by a 4-tuple* $G=(N,A_{M},A_{S},U)$*, where:*

- N={malware, system} *is a set of players;*
- $A_{M}=\{Infect\;(I),\;Non-infect\;(\phi )\}$ *is a set of actions performed by player* malware*;*
- $A_{S}=\{Defend\;(D),\;Non-defend\;(\phi )\}$ *is a set of actions performed by player* system*;*
- $U:A_{M}\times A_{S}\mapsto \mathbb{R}$ *is a payoff matrix.*

Let $\rho_{I}$ and $\rho_{\phi }$ be the probabilities that player *malware* adopts actions *Infect* and *Non-infect*, respectively. Let $\delta_{D}$ and $\delta_{\phi }$ be the probabilities that player *system* adopts actions *Defend* and *Non-defend*, respectively. Accordingly, infection strategy ρ and defense strategy δ are mixed strategies $\left(\rho_{I},\rho_{\phi }\right)$ and $\left(\delta_{D},\delta_{\phi }\right)$, which represent the probability distributions over action sets $A_{M}$ and $A_{S}$, respectively. Both of them certainly satisfy $\rho_{I}+\rho_{\phi }=1$ and $\delta_{D}+\delta_{\phi }=1$. Actually, the infection probability $\rho_{I}$ describes the degree of infection for a sensor node (equivalently, the aggressiveness of player *malware* targeting a sensor node). The larger $\rho_{I}$ is, the greater the probability of action *Infect* and, hence, the larger the corresponding infection rate for a sensor node.

Now we consider the payoff matrix to explore *malware*’s motivation. For simplicity, we denote the worth of a sensor node by ω, where ω>0. Actually, ω is equivalent to a degree of damage such as the loss of sensed data, loss due to compromise, and so on. If player *malware* infects successfully, it will obtain payoff ω and its opponent will obtain payoff −ω. On the contrary, if player *system* succeeds in defense, its payoff is ω because it has protected a sensor node worth ω and player *malware* will be penalized by ω. However, no IDS can entirely detect all current and future malware: all IDSes have true positive rates and false positive rates. Next, we consider these two rates as defining the payoffs of *malware* and *system* and we let α and β be the true positive rate and the false positive rate of the WSN IDS, respectively. We also let $c_{I}$ and $c_{D}$ be the cost of player *malware* infecting a susceptible sensor node and player *system* detecting the *malware*’s infection, respectively. Obviously, there are four possible payoffs constructing the payoff matrix, since each of *malware* and *system* has two possible actions. For the action profile (*Infect*, *Defend*), player *malware* will obtain gain (1−α)ω from detection failure as well as loss αω from being detected successfully and loss $c_{I}$ from adopting action *Infect*. Thus, the payoff of player *malware*, $u_{ID}^{I}$, is:

(2) $$u_{ID}^{I}=(1-\alpha )\omega -\alpha \omega -c_{I}=(1-2\alpha )\omega -c_{I}$$

On the other hand, player *system* obtains gain αω as well as loses (1−α)ω from detection failure, βω from false positive detection, and $c_{D}$ from detecting *malware*’s infection. Thus, the payoff of player *system*, $u_{ID}^{S}$, is:

(3) $$u_{ID}^{S}=\alpha \omega -(1-\alpha )\omega -\beta \omega -c_{D}=(2\alpha -1-\beta )\omega -c_{D}$$

For the action profile (*Infect*, *Non*-*defend*), player *malware* obtains gain λω from successful infection and loses $c_{I}$ from adopting action *Infect*. Thus, the payoff of player *malware*, $u_{I\phi }^{I}$, is:

(4) $$u_{I\phi }^{I}=\lambda \omega -c_{I}$$

whereas the payoff of player *system*, $u_{I\phi }^{S}$, is:

(5) $$u_{I\phi }^{S}=-\lambda \omega$$

For the action profile (*Non*-*infect*, *Defend*), the payoff of player *malware*, $u_{\phi D}^{I}$, is:

(6) $$u_{\phi D}^{I}=0$$

whereas the payoff of player *system*, $u_{\phi D}^{S}$, is:

(7) $$u_{\phi D}^{S}=-\beta \omega -c_{D}$$

Finally, for the action profile (*Non-infect*, *Non-defend*), since neither player *malware* nor player *system* can obtain any gain or produce any loss, the payoff of player *malware*, $u_{\phi \phi }^{I}$, is:

(8) $$u_{\phi \phi }^{I}=0$$

and the payoff of player *system*, $u_{\phi \phi }^{S}$, is:

(9) $$u_{\phi \phi }^{S}=0$$

Table 1 summizes our defined payoff matrix.

The objective of player *malware* is to maximize its expected infection utility, whereas the objective of player *system* is to minimize its expected defense utility. This objective can be achieved by solving the mixed-strategy Nash Equilibrium (NE) of the strategic game.

**Theorem 1.**  *In the SMIG, the optimal probability of player malware choosing action Infect is:*

(10) $$\rho_{I}^{*}=\frac{\beta \omega +c_{D}}{(\lambda +2\alpha -1)\omega }$$

**Proof:**  Under player *malware*’s mixed strategy, player *system*’s expected payoffs for choosing actions *Defend* and *Non-defend* are:

(11) $$E_{S}(Defend)=\rho_{I}((2\alpha -1-\beta )\omega -c_{D})+(1-\rho_{I})(-\beta \omega -c_{D})$$

and:

(12) $$E_{S}(Non-defend)=\rho_{I}(-\lambda \omega )+(1-\rho_{I})\cdot 0=-\rho_{I}\lambda \omega$$

respectively. From the indifference between actions *Defend* and *Non-defend* under the optimal mixed strategy of player *system*, we obtain:

(13) $$E_{S}(Defend)=E_{S}(Non-defend)$$

Therefore, the optimal probability of player *malware* choosing action *Infect* is:

$$\rho_{I}^{*}=\frac{\beta \omega +c_{D}}{(\lambda +2\alpha -1)\omega }$$

 □

Obtaining the optimal infection probability $\rho_{I}^{*}$ means that we acquire the indication of the expected infection behavior of player *malware* for WSNs under epidemic-malware propagation. In other words, malware will choose action *Infect* with probability $\rho_{I}^{*}$. When following $\rho_{I}^{*}$, player *malware* has no reason to adjust its strategy as it has maximized its expected utility from the infection regardless of the success of its actions.

## 5. Reliability Evaluation Method

### 5.1. Evaluating the Reliability of a Sensor Node

The reliability of WSNs represents the probability that sensor nodes continue to perform tasks such as data sensing, transmission, and fusion over a particular period of time under stated conditions. In general, MTTF and MTBF (Mean Time between Failures) are typical ways to evaluate the reliability of pieces of hardware or other technology. Here, MTTF refers to the length of time that a device is expected to last in operation, whereas MTBF is the average elapsed time between a device’s failures in operation. The difference between two terms is that MTTF is used for non-repairable devices, whereas MTBF is used for devices that can be repaired and returned to operation. In this work, we concentrate on WSNs where sensor nodes can hardly be repaired upon failure and we use MTTF for evaluating the reliability of a sensor node.

We denote ℰ as the discrete state space where:

(14) $$ℰ=\{S,\;\overset{⌢}{S},\;I,\;\overset{⌢}{I},\;R,\;\overset{⌢}{R},\;D\}$$

as illustrated in Figure 1. Let:

(15) $$Y(t)=[Y_{S}(t){\text{ Y}}_{\overset{⌢}{S}}(t){\text{ Y}}_{I}(t){\text{ Y}}_{\overset{⌢}{I}}(t){\text{ Y}}_{R}(t){\text{ Y}}_{\overset{⌢}{R}}(t){\text{ Y}}_{D}(t)]$$

be the state probability vector, where $Y_{x}(t)$ denotes the probability that a sensor node is in state x, x∈ℰ, at time t. Let P be the 7×7 state transition matrix consisting of element $p_{ij}$, i, j∈ℰ. We find the state equation of a sensor node as:

(16) $$\frac{dY(t)}{dt}=Y(t)P$$

We find the steady-state probability vector that is independent of Y(0) (*i.e.*, the initial state):

(17) $$Y=[Y_{S}{\text{ Y}}_{\overset{⌢}{S}}{\text{ Y}}_{I}{\text{ Y}}_{\overset{⌢}{I}}{\text{ Y}}_{R}{\text{ Y}}_{\overset{⌢}{R}}{\text{ Y}}_{D}]$$

by solving the system of seven equations where six of the seven equations are from:

(18) YP=0

and the seventh equation is:

(19) $$\sum_{i\in ℰ}Y_{i}=1$$

Next, we compute a sensor node’s MTTF from the steady-state probability vector. The discrete state space ℰ can be split into two disjoint sets ${ℰ}_{Use}$ and ${ℰ}_{Disuse}$, where:

(20) $${ℰ}_{Use}=\{S,\;R\}$$

and:

(21) $${ℰ}_{Disuse}=\{\overset{⌢}{S},\;I,\;\overset{⌢}{I},\;\overset{⌢}{R},\;D\}$$

denote the set of usable and unusable states, respectively. Correspondingly, the state transition matrix P can be rewritten as:

(22) $$P=\left[\begin{array}{cc} P_{1} & P_{2}\\ P_{3} & P_{4} \end{array}\right]$$

where:

(23) $$P_{1}=\left[\begin{array}{cc} p_{SS} & p_{SR}\\ p_{RS} & p_{RR} \end{array}\right]$$

(24) $$P_{2}=\left[\begin{array}{cc} p_{S\overset{⌢}{S}} & \begin{array}{cc} \begin{array}{ccc} p_{SI} & p_{S\overset{⌢}{I}} & p_{S\overset{⌢}{R}} \end{array} & p_{SD} \end{array}\\ p_{R\overset{⌢}{S}} & \begin{array}{ccc} \begin{array}{cc} p_{RI} & p_{R\overset{⌢}{I}} \end{array} & p_{R\overset{⌢}{R}} & p_{RD} \end{array} \end{array}\right]$$

(25) $$P_{3}=\left[\begin{array}{cc} p_{\overset{⌢}{S}S} & p_{\overset{⌢}{S}R}\\ p_{IS} & p_{IR}\\ p_{\overset{⌢}{I}S} & p_{\overset{⌢}{I}R}\\ p_{\overset{⌢}{R}S} & p_{\overset{⌢}{R}R}\\ p_{DS} & p_{DR} \end{array}\right]$$

and:

(26) $$P_{4}=\left[\begin{array}{ccccc} p_{\overset{⌢}{S}\overset{⌢}{S}} & p_{\overset{⌢}{S}I} & p_{\overset{⌢}{S}\overset{⌢}{I}} & p_{\overset{⌢}{S}\overset{⌢}{R}} & p_{\overset{⌢}{S}D}\\ p_{I\overset{⌢}{S}} & p_{II} & p_{I\overset{⌢}{I}} & p_{I\overset{⌢}{R}} & p_{ID}\\ p_{\overset{⌢}{I}\overset{⌢}{S}} & p_{\overset{⌢}{I}I} & p_{\overset{⌢}{I}\overset{⌢}{I}} & p_{\overset{⌢}{I}\overset{⌢}{R}} & p_{\overset{⌢}{I}D}\\ p_{\overset{⌢}{R}\overset{⌢}{S}} & p_{\overset{⌢}{R}I} & p_{\overset{⌢}{R}\overset{⌢}{I}} & p_{\overset{⌢}{R}\overset{⌢}{R}} & p_{\overset{⌢}{R}D}\\ p_{D\overset{⌢}{S}} & p_{DI} & p_{D\overset{⌢}{I}} & p_{D\overset{⌢}{R}} & p_{DD} \end{array}\right]$$

Likewise, we split the steady-state probability vector into two disjoint parts $Y_{Use}$ and $Y_{Disuse}$, where:

(27) $$Y_{Use}=[Y_{S}{\text{ Y}}_{R}]$$

and:

(28) $$Y_{Disuse}=[Y_{\overset{⌢}{S}}{\text{ Y}}_{I}{\text{ Y}}_{\overset{⌢}{I}}{\text{ Y}}_{\overset{⌢}{R}}{\text{ Y}}_{D}]$$

According to the method provided in [53] to compute the MTTF from a Markov process, we find the MTTF of a sensor node η as:

(29) $$\eta =Y_{Use}(0){\left(-P_{1}\right)}^{-1}I$$

where $Y_{Use}(0)$ denotes the initial usable state probability vector (*i.e.*, with t=0) computed as:

(30) $$Y_{Use}(0)=\frac{Y_{Use}}{Y_{Use}I}$$

and I denotes a column vector of two ones:

(31) $$I={\left[1\;1\right]}^{-1}$$

Let $Reliability_{node}(t)$ be the reliability of a sensor node at time t. We assume that all sensor nodes, due to their similarity, have the same MTTF. From reliability theory, we find that:

(32) $$Reliability_{node}(t)=\exp(-\frac{1}{\eta }t)$$

### 5.2. Evaluating Reliability of a Clustered WSN

**Figure 2:**
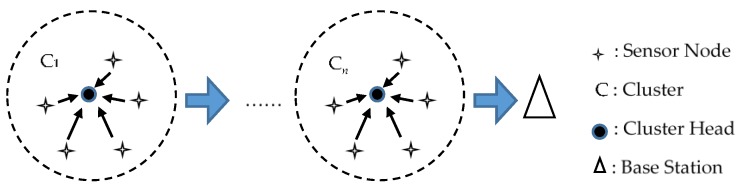
Topology of a clustered WSN, where *n* denotes the number of clusters in a route.

We aim to perform reliability evaluations for clustered WSNs due to their popularity. Figure 2 illustrates the topology of a clustered WSN. Coordinating cluster heads (CHs) control the topology where each CH guides a different cluster of sensor nodes. Such an architecture leads to a two-tier hierarchy where the upper tier comprises CHs and the lower tier comprises sensor nodes. Sensor nodes in specific regions transmit their sensed data to the responsible CH that manages the nodes. The responsible CH collects the data, which are transmitted to the single base station via other CHs. In this way, we can relate each cluster to a parallel system and each set of clusters to a serial system. Therefore, any route from a sensor node to the base station can be naturally regarded as a serial-parallel path. Since there are various routes through which sensed data can be transferred, a clustered WSN can be mapped correspondingly to a parallel-serial-parallel system where each sensor node fails independently. Each cluster operates if at least one of its sensor nodes works normally. However, for any route, all of its clusters must work normally for proper operation. As a result, we can evaluate the reliability of a clustered WSN based on the MTTF of a sensor node from the perspective of classical reliability theory.

Since each cluster is a parallel system, the reliability of a cluster at time t, $Reliability_{cluster}(t)$, can be computed as:

(33) $$Reliability_{cluster}(t)=1-\prod_{node\in cluster}\left(1-Reliability_{node}(t)\right)$$

Moreover, any route composed of clusters is a serial system, and thus the reliability of a route at time t, $Reliability_{route}(t)$, can be computed as:

(34) $$Reliability_{route}(t)=\prod_{cluster\in route}Reliability_{cluster}(t)$$

Finally, a clustered WSN composed of available routes is a parallel system. Therefore, the reliability of a clustered WSN at time t, Reliability(t), is:

(35) $$Reliability(t)=1-\prod_{route}\left(1-Reliability_{route}(t)\right)$$

So far, we have developed a method of reliability evaluation for clustered WSNs under epidemic-malware propagation conditions. In practice, we suggest using the proposed method in off-line mode, since performing on-line reliability evaluation for clustered WSNs is very difficult. First, we define our strategic game mathematically and solve it analytically. We integrate the results of the game with the transition probability upon infection of a sensor node from which we compute the MTTF of a sensor node. As soon as the topology of a clustered WSN is determined based on the actual requirements, we can find the number of sensor nodes in a cluster, the number of clusters in a route, and the number of routes in a clustered WSN. Based on these numbers, we can compute the reliability of a clustered WSN from Equation (35). In fact, our way is popular in the field of using the game theoretical approaches, which is easy to realize.

## 6. Numerical Results

### 6.1. Illustrating Influence of α and β

With MATLAB R2010b, we explore how the optimal infection probability and the MTTF of a sensor node depend on the parameters of the true positive rate (*i.e.*, the detection rate) and the false positive rate (*i.e.*, the false alarm rate). The parameters of the strategic game are ω=50, $c_{I}=20$, $c_{D}=10$, and λ=0.5. Note that we can attain similar trends if the parameters are changed. However, specific values will be correspondingly changed.

**Figure 3:**
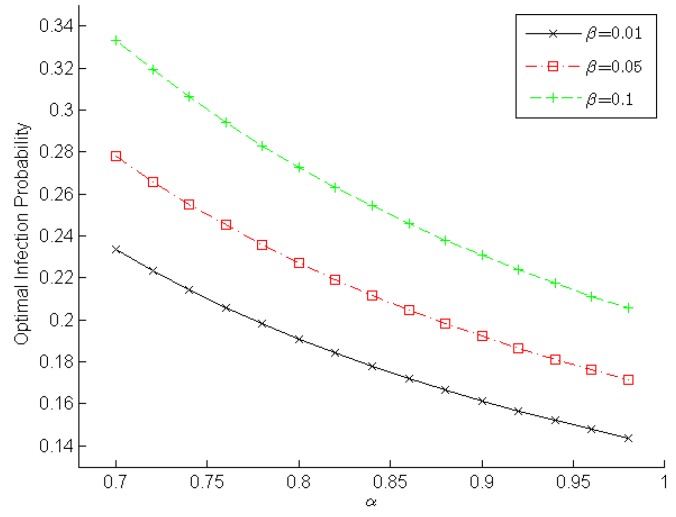
Optimal infection probability in terms of the true positive rate.

**Figure 4:**
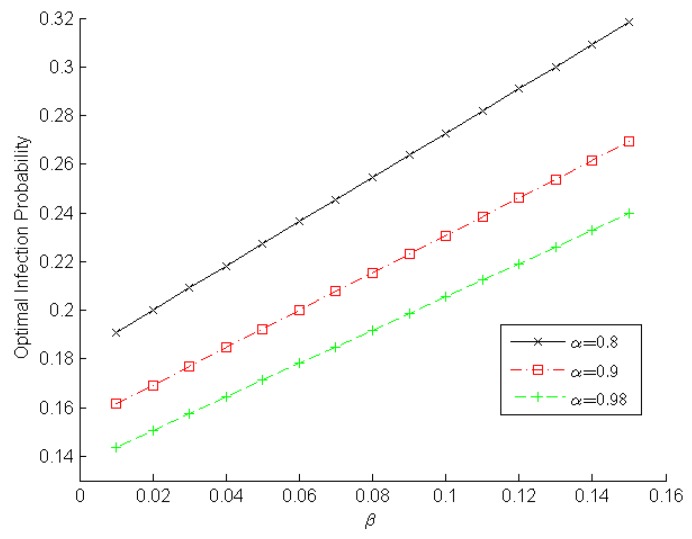
Optimal infection probability in terms of the false positive rate.

Figure 3 and Figure 4 demonstrate the changing optimal infection probabilities that player *malware* adopts according to α and β, which reveals the *malware*’s intention. Obviously, a higher detection rate and a lower false-alarm rate can help a WSN IDS detect malware. Therefore, player *malware* will choose its optimal strategy to lower the infection probability in order to minimize the loss arising from IDS detection. As shown in Figure 3, the optimal infection probability decreases gradually when the true positive rate increases slowly from 0.7 to 0.98. Moreover, a lower false positive rate results in a lower infection probability. For example, when α=0.88 in Figure 3, the optimal infection probabilities are ~0.1667, ~0.1984, and ~0.2381 for β=0.01, β=0.05, and β=0.1, respectively. We observe in Figure 4 the optimal infection probability increases gradually when the false positive rate increases slowly from 0.01 to 0.15. Furthermore, a higher detection rate leads to a lower infection probability. For example, when β=0.1 in Figure 4, the optimal infection probabilities are ~0.2727, ~0.2308, and ~0.2055 for α=0.8, α=0.9, and α=0.98, respectively. These experimental results indicate that the true positive rate should be increased and the false positive rate should be decreased in order to decrease the infection probability adopted by player *malware* and to enhance a sensor node’s reliability.

**Figure 5:**
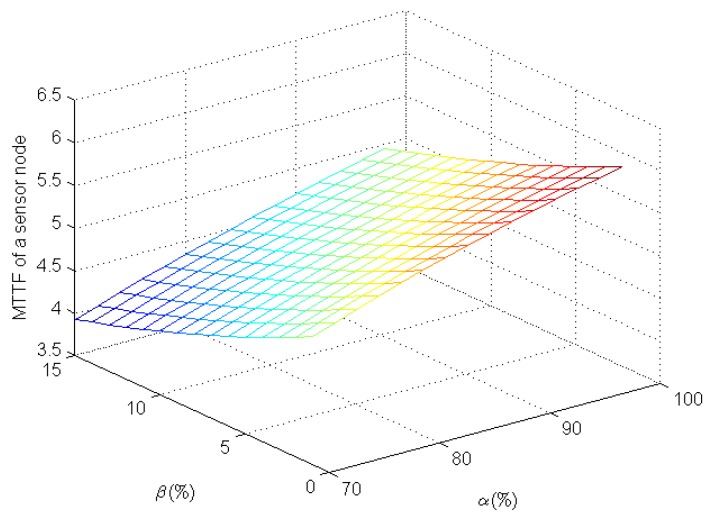
A sensor node’s MTTF in terms of the true positive rate and the false positive rate.

Figure 5 shows the MTTF of a sensor node under the epidemic-malware propagation scenario in terms of α and β. As the true positive rate increases and the false positive rate decreases, it is easy for player *system* to detect infected sensor nodes, increasing their MTTFs. From Figure 5, as we expect, the MTTF increases slowly when the true positive rate increases gradually from 70% to 98%. There is a similar tendency when the false positive rate decreases from 15% to 1%. Smaller decreases of the false positive rate increase the MTTF for a sensor node more than the same decreases of the true positive rate. For example, the MTTF of a sensor node increases from ~4.6366 to ~5.7219 (an increase of ~23.41%) as β drops from 15% to 1% when α=88%. However, when β=10%, the MTTF of a sensor node increases from ~4.2487 to ~5.0350 (an increase of ~18.51%) as α increases from 70% to 90%. These results indicate that we should further reduce the false positive rate while improving IDSes for WSNs in order to increase a sensor node’s MTTF.

### 6.2. Validating the Method of Reliability Evaluation for a Clustered WSN

**Figure 6:**
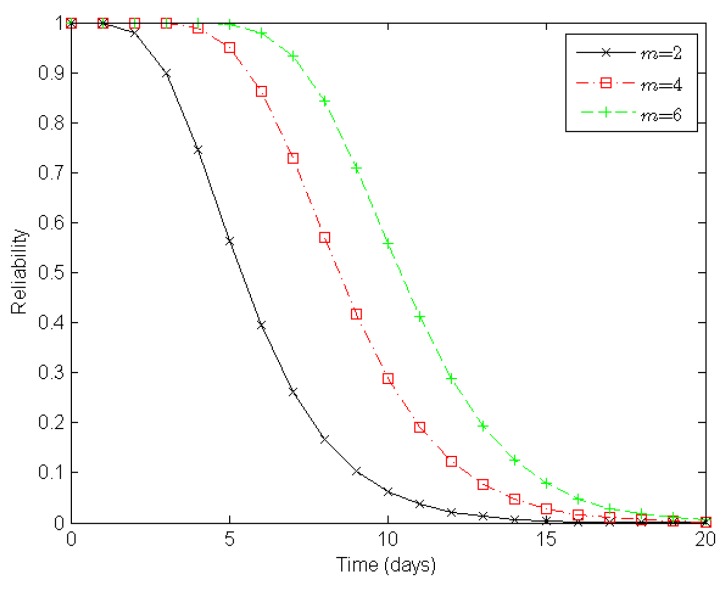
Reliability of a clustered WSN when *m* = 2, *m* = 4, and *m* = 6, respectively. Here, *m* denotes the number of sensor nodes in a cluster. There are four clusters in a route and four routes in a clustered WSN.

**Figure 7:**
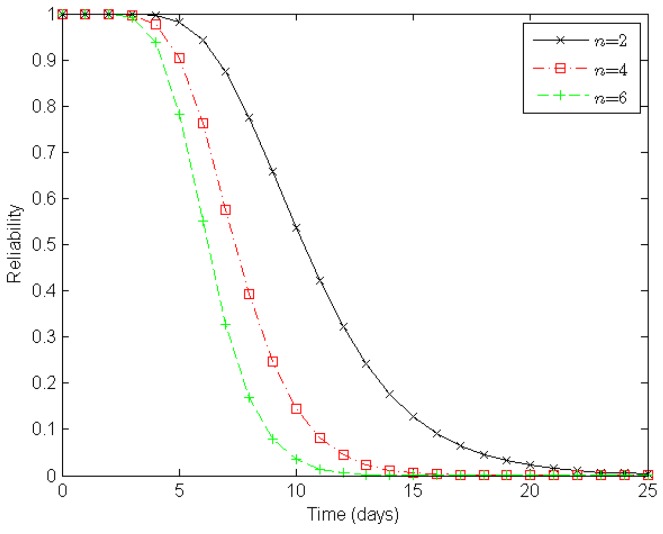
Reliability of a clustered WSN when *n* = 2, *n* = 4, and *n* = 6, respectively. Here, *n* denotes the number of clusters in a route. There are four sensor nodes in a cluster and four routes in a clustered WSN.

**Figure 8:**
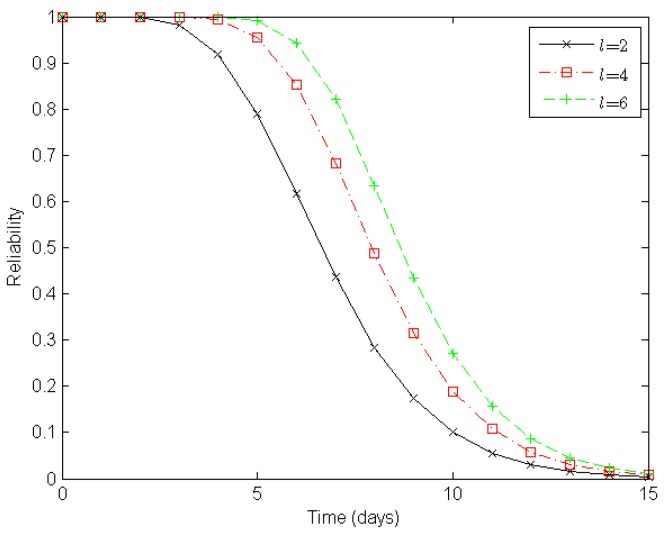
Reliability of a clustered WSN when *l* = 2, *l* = 4, and *l* = 6, respectively. Here, *l* denotes the number of routes in the clustered WSN. There are four sensor nodes in a cluster and four clusters in a route.

Next, from the perspective of a clustered WSN, we evaluate its reliability according to the number of sensor nodes in a cluster, the number of clusters in a route, and the number of routes in a clustered WSN, as illustrated in Figure 6, Figure 7 and Figure 8, respectively.

Figure 6 illustrates varying degrees of reliability for a clustered WSN when there are two, four, and six sensor nodes in a cluster, respectively. When both the number of clusters in a route and the number of routes in a clustered WSN are static, the reliability of the WSN increases with the number of sensor nodes in a cluster. With two, four, and six nodes in a cluster, it takes about six, nine, and eleven days, respectively, to reduce the reliability of a clustered WSN to 0.5 under the epidemic-malware propagation scenario.

Figure 7 illustrates varying degrees of reliability for a clustered WSN when there are two, four, and six clusters in a route, respectively. When both the number of sensor nodes in a cluster and the number of routes in a clustered WSN are static, the reliability of the WSN decreases with the number of clusters in a route. With two, four, and six clusters in a route, it takes about eleven, eight, and seven days, respectively, to reduce the reliability of a clustered WSN to 0.5 under the epidemic-malware propagation scenario.

Figure 8 illustrates varying degrees of reliability for a clustered WSN when there are two, four, and six routes in a clustered WSN, respectively. When both the number of sensor nodes in a cluster and the number of clusters in a route are static, the reliability of the WSN increases with the number of routes in the clustered WSN. With two, four, and six routes in the clustered WSN, it takes about seven, eight, and nine days, respectively, to reduce the reliability of the clustered WSN to 0.5 under the epidemic-malware propagation scenario.

In summary, the experimental results shown in Figure 6, Figure 7 and Figure 8 indicate that deploying more redundant sensor nodes in a cluster, deducing the clusters along constructed routes, and providing more available routes all help improve the reliability of a clustered WSN, which accords with our expectations.

## 7. Conclusions

We have performed reliability analysis on clustered WSNs under the epidemic-malware propagation scenario and developed a corresponding measure of reliability evaluation in order to establish a kind of highly reliable WSN. We have determined how to relate the intent of malware infection to the randomness of CTMCs using a strategic game that can predict malware’s infection behavior. We have proposed the MTTF to reflect the reliability of a sensor node and we have regarded clustered WSNs as a parallel-serial-parallel system. Using this approach, we have obtained equations to compute the reliability of a cluster, a route, and a clustered WSN, respectively. As a result, we have provided a foundation for the mechanism of reliability evaluation for susceptible WSNs. Our experiments have shown the importance of reducing the false positive rate rather than the true positive rate in order to increase MTTFs for susceptible sensor nodes. We have also validated the efficacy of our proposed measure of reliability evaluation for susceptible WSNs.

We have assumed that the topology of a clustered WSN only consists of a single sink node; however, actual clustered WSNs may have several sink nodes. Furthermore, the topology of a clustered WSN may be changed once mobile sensor nodes are introduced. Under these circumstances, the equation to compute the reliability of the clustered WSN will be more complicated. Providing such a reliability evaluation method is an interesting research direction when the assumption is relaxed. Moreover, providing measures of availability, dependability, and survivability for WSNs under malware propagation is another interesting direction.

## Figures and Tables

**Table 1:** The payoff matrix of the SMIG.

	*Defend*	*Non-Defend*
*Infect*	$(1-2\alpha )\omega -c_{I}$, $(2\alpha -1-\beta )\omega -c_{D}$	$\lambda \omega -c_{I}$, −λω
*Non-infect*	0, $-\beta \omega -c_{D}$	0, 0
